# Characteristics of lymphoedema, in particular midline lymphoedema, after treatment for prostate cancer: a retrospective study

**DOI:** 10.1186/s12894-024-01533-5

**Published:** 2024-09-04

**Authors:** Charlotte Van Calster, Wouter Everaerts, Inge Geraerts, An De Groef, An-Kathleen Heroes, Tessa De Vrieze, Ceyhun Alar, Nele Devoogdt

**Affiliations:** 1 Department of Rehabilitation Sciences, KU Leuven - University of Leuven, Leuven, Belgium; 2 Department of Urology, University Hospitals Leuven, Leuven, Belgium; 3 Department of Cellular and Molecular Medicine, KU Leuven - University of Leuven, Leuven, Belgium; 4 Department of Physical Medicine and Rehabilitation, University Hospitals Leuven, Leuven, Belgium; 5 Department of Rehabilitation Sciences and Physiotherapy, University of Antwerp, MOVANT, Wilrijk, Belgium; 6 Institute for Single Cell Omics (LISCO), KU Leuven – University of Leuven, Leuven, Belgium; 7 Department of Human Genetics, KU Leuven – University of Leuven, Leuven, Belgium; 8 Center for Lymphoedema, University Hospitals Leuven, Leuven, Belgium

**Keywords:** Lymphedema, Genital, Leg, Prostate cancer, Prostatectomy, Lymph node dissection

## Abstract

**Background:**

Patients undergoing treatment for prostate cancer may develop lymphoedema of the midline region. This has a substantial impact on a patient’s quality of life and its diagnosis is often delayed or missed. Therefore, the purpose of this study is to compare the characteristics of patients with leg and midline lymphoedema to patients with only leg lymphoedema.

**Methods:**

We retrospectively collected patient-, cancer-, lymphoedema- and lymphoedema treatment-related data of 109 men with lymphoedema after treatment for prostate cancer. First, 42 characteristics were compared between both groups. Second, factors predicting presence of midline lymphoedema were explored by multivariable analyses.

**Results:**

The mean age of the patients with lymphoedema was 68 ( ±7) years and mean BMI is 28 (±4) kg/m^2^. Median duration of lymphoedema before the first consultation was 27 (9;55) months. Based on univariable analyses, patients with leg and midline lymphoedema had more frequently upper leg lymphoedema (89% (31/35) vs. 69% (51/74), *p* = 0.026), skin fibrosis (34% (12/35) vs. 16% (12/74), *p* = 0.034) and lymphatic reconstructive surgery (9% (3/35) vs. 0% (0/71), *p* = 0.020) than patients with only leg lymphoedema. Additionally, patients with leg and midline lymphoedema reported less frequently lower leg lymphoedema (77% (27/35) vs. 95% (70/74), *p* = 0.007). Based on the multivariable analysis, not having lower leg lymphoedema, skin fibrosis, performing self-bandaging and self-manual lymphatic drainage appear to be predictors for having midline lymphoedema.

**Conclusions:**

If patients with lymphoedema after prostate cancer do not have lower leg lymphoedema, have skin fibrosis, perform self-bandaging or self-manual lymphatic drainage, they possibly have midline lymphoedema.

**Supplementary Information:**

The online version contains supplementary material available at 10.1186/s12894-024-01533-5.

## Introduction

Prostate cancer (PCa) ranks the second most common cancer in men worldwide [1]. International guidelines of 2022 recommend considering a radical prostatectomy accompanied by an extended lymph node dissection in patients diagnosed with high-risk PCa as well as intermediate-risk PCa, when the risk of lymph node metastases exceeds 5% [2].

Although an extended lymph node dissection is considered the most accurate staging method for detecting microscopic lymph node involvement, the therapeutic benefit of the procedure is still debated [2, 3]. Moreover, performing an extended lymph node dissection increases the morbidity of the surgical procedure, with higher complication rates associated with more extended dissections [3–5]. Among these complications, lymphoedema is noteworthy, alongside pelvic lymphocele, deep venous thrombosis and pulmonary embolisms [5, 6]. Lymphoedema is characterized by swelling of a body part because of interstitial fluid accumulation due to insufficient lymphatic drainage. Lymphoedema can be classified into stage 1 (reversible), stage 2 (irreversible; pitting evolving to non-pitting adipose tissue) and stage 3 (elephantiasis; non-pitting adipose tissue and fibrosis) [7]. Lymphoedema has a substantial impact on a person’s quality of life, with a negative correlation between a patient’s quality of life and both presence and volume of cancer-related lymphoedema [8].

In the case of a pelvic lymph node dissection, the legs and midline are at risk for developing lymphoedema. Leg lymphoedema means that the upper leg, lower leg, foot or a combination can be swollen. Midline lymphoedema encompasses genital (the penis and/or scrotum) and/or supra pubic lymphoedema. A recent systematic review conducted by Clinckaert et al. reported leg and midline lymphoedema rates of 0–14% and 0–1%, respectively. Notably, these rates increase dramatically when patients receive pelvic radiotherapy after pelvic lymph node dissection (to 18–29% for leg lymphoedema and 2–22% for midline lymphoedema) [9]. As reliable and valid tools are missing, detection of midline lymphoedema is based on palpation and visual inspection of the midline region [10].

Importantly, the majority of existing lymphoedema literature primarily focuses on upper limb lymphoedema after breast cancer and lower limb lymphoedema after gynecological cancer. As a consequence, literature on detection and presence of midline lymphoedema after PCa treatment is scarce. Furthermore, in clinical practice, patients with midline lymphoedema often experience embarrassment, which leads them to avoid discussing this problem with their therapist. Consequently, therapists may refrain from addressing the issue and often do not inquire about swelling in this sensitive area [11]. This probably leads to missing of the detection of midline lymphoedema. Therefore, it is interesting to know which other characteristics of the PCa patient with lymphoedema are associated with the presence of midline lymphoedema.

The aim of the present retrospective study is to compare the characteristics of patients between a group with leg and midline lymphoedema and a group with only leg lymphoedema through univariable and multivariable analyses.

## Materials and methods

This retrospective study has obtained approval from the Ethical Committee of UZ/ KU Leuven (S66167).

### Patients

Data was collected from newly referred patients to the center for lymphoedema at the University Hospitals Leuven, campus Pellenberg between May 2018 and January 2022.The inclusion criteria were: (1) patients who underwent local therapy for prostate cancer, (2) patients with lymphoedema of the legs and/or midline region and (3) patients with at least 70% data completeness in the source document. The diagnosis was clinically established through inspection (location of the oedema and skin changes), palpation (pitting, tissue fibrosis) and collecting information about the patient history. Volume measurements of both legs and feet were performed and presence of lymphoedema was defined as 5% or more volume difference between both legs and feet. In case of bilateral lymphoedema and less than 5% volume difference, a lymphoscintigraphy was performed to confirm the diagnosis of lymphoedema.

### Data collection and processing

All data was collected using a structured source document designed with Microsoft Infopath Designer 2013^Ⓡ^. These source documents are used in routine clinical care at the center for lymphoedema and are incorporated in the Electronic Health Records (HER) system. The forms consist of following sheets: anamnesis (during which complaints, duration of lymphoedema, infections, medical history and relevant factors are questioned), inspection and palpation, clinical investigation, diagnosis, followed by treatment and treatment plan.

In February 2022, source documents of all patients with diagnosis of prostate cancer visiting the center for lymphoedema between May 2018 and January 2022, were exported. Data were pseudonymised and transferred to an Excel^Ⓡ^ datasheet. There was data from 544 consultations in 135 individual patients. First, data from the first consultation of a specific patient was selected. In case this form did not have enough information, the form of the second consultation was selected. Second, stable outcomes of interest (including medical history, oncological hormonal therapy, oncological radiotherapy, onset of lymphoedema after surgery for prostate cancer or gland removal), were added in case of missing data for a given patient. Third, only patients of which a minimum of 70% of the necessary outcomes were completed were included. Fourth, the presence of lymphoedema at the level of the leg and/or midline region was verified. In case lymphoedema was not present, the record of the patient was removed. Finally, all 42 variables of our interest, were extracted from the Excel datasheet. Additional file 1 gives an overview of all variables and how they were calculated from raw data. All variables were categorized into four groups: (1) patient-related variables, (2) cancer-related variables, (3) lymphoedema-related variables and (4) lymphoedema treatment-related variables.

### Data analysis

All data was retrospectively analysed with SPSS^Ⓡ^ (IBM) version 28.0.1.1 (14).

Descriptive statistics were used to outline the characteristics of patients with lymphoedema.

Univariable analyses were performed to compare the characteristics of patients (see Table 1) with or without midline oedema. For normal and not normal distributed continuous data an independent T-test and Mann-Whitney U test was used, respectively. A Chi^2^ test was used for discontinuous data. A p-value of ≤ 0.050 indicated statistical significance.

Next, multivariable analyses were conducted, beginning with an assessment of missing data. Since less than 70% of the 109 cases had complete data, multiple imputation was employed. This involved creating several copies of the dataset, replacing missing values with imputed values drawn from a predictive distribution based on available observed data. This resulted in a new dataset with 10 imputations. Binary logistic regression was then performed on this pooled dataset, with the presence of midline lymphoedema chosen as the dependent variable. Independent variables with a significance level of *p* < 0.100 in the univariable analyses were included in the model. Additionally, variables of interest commonly found in literature and present in at least 10 out of 109 patients were added. A forward method was utilized, where model expansion at each step was guided by p-values derived from Rubin’s rule, a statistical approach for obtaining p-values from imputed datasets [12]. The criteria for entry and removal were set at *p* ≤ 0.050 and *p* ≥ 0.100, respectively. Odds ratios and corresponding 95% confidence intervals from the binary logistic model were reported along with their significance levels.

Finally, the c-index with 95% confidence interval was calculated after multiple imputation. The c-index is also known as the Area under the Receiver Operating Characteristic Curve (AUC-ROC). It is a metric which is used to evaluate the performance of predictive models. Which in this study means the ability of the model to make a distinction between patients with leg and midline lymphoedema and patients with only leg lymphoedema. To enhance the model’s robustness a cross-validation technique was applied in which the data was randomly split into 80% for training the model and 20% for testing its performance. We only used the original, raw data instead of the imputed dataset. This decision was made because the model’s variables have binary outcomes and were not subject to imputation, ensuring the avoidance of artificial duplicates.

## Results

In total, data of 109 patients was available for analyses.

### Patient characteristics

See Table 1 for the patient-, cancer-, lymphoedema- and lymphoedema-related characteristics of the study sample (*n* = 109).

Mean age was 68 (±7) years with a mean BMI of 28 (±4) kg/m^2^. The majority of the men were non-smokers (87%) and half of them practiced sports (59%). Comorbidities (kidney disease, diabetes, thrombosis, cardiac- and thyroid disease) were infrequent (between 4% and 17%), illustrating patient selection for prostate cancer surgery.

Almost all (93%) participants underwent lymph node dissection. Radiotherapy was given to 46% of patients and hormonal therapy applied to 33% of patients.

The median duration of lymphoedema was 27 months (9;55) at the time of the first consultation at the center for lymphoedema. Median onset of lymphoedema after surgery was 3 months (0;10). 59% experienced lymphoedema of the foot, 89% of the lower leg and 75% of the upper leg. Sixty-five per cent had lymphoedema affecting the entire leg except the foot and 32% suffered from midline lymphoedema. Of these 35 patients with leg and midline lymphoedema, 14 (40%) had penile oedema, 19 (54%) scrotum oedema and 29 (83%) supra pubic oedema. Palpation for evaluating lymphoedema indicated that most patients (81%) had pitting oedema and soft (not hard) oedema (78%). 6% of the patients also had a wound at the leg. The most common stage of lymphoedema was stage 2a (58%), followed by 29 patients (28%) with stage 1 lymphoedema and 15 patients (14%) with stage 2b lymphoedema.

Most patients (79%) wore compression stocking(s) with a median of 30 days per month (13;30). Fifty-five per cent of the patients applied skincare with a median of 4 days (0;16) per week. Most patients (58%) did not exercise at home, 50% indicated going to a physiotherapist and 41% received manual lymphatic drainage. Only 3 patients (2%) underwent surgery for the treatment of lymphoedema with all 3 patients undergoing reconstructive lymphatic surgery and no liposuction.

### Comparison between the group with and without midline oedema

#### Univariable analyses

An overview of the comparison between the patients with leg and midline lymphoedema (*n* = 35) and the patients with only leg lymphoedema (*n* = 74) is presented in Table 1.

Patient’s age and BMI and other patient-related variables were not significantly different between both groups.

Cancer-related variables were not significantly different between both groups.

The rates of lymphoedema in the lower and upper leg were statistically different between the two groups. Twenty-seven patients of the leg and midline lymphoedema group (77%) and 70 patients of the leg lymphoedema group (95%) had lymphoedema of the lower leg (*p* = 0.007), whereas 31 patients with leg and midline oedema (89%) and 51 patients (69%) with only leg lymphoedema suffered from lymphoedema in the upper leg (*p* = 0.026). Significantly more patients with leg and midline lymphoedema had fibroses (34%) compared to patients with only leg lymphoedema (16%) (*p* = 0.034). None of the patients with leg and midline lymphoedema had wounds (*p* = 0.029) whereas 6 (8%) patients with leg lymphoedema had wounds.

Three patients (9%) of the leg and midline lymphoedema group underwent lymphatic surgery while none of the patients of the leg lymphoedema group (*p* = 0.020). More patients with leg and midline lymphoedema performed self-manual lymphatic drainage compared to the group with leg lymphoedema (18% vs. 4%; *p* = 0.045) and also more patients from the leg and midline lymphoedema group performed self-bandaging (33% vs. 15%; *p* = 0.082). No other differences were found.

**Table 1:** Univariable analyses of the whole sample and a comparison between the two groups

Variable	All patients (n = 109)		Patients with leg + midline lymphoedema (n = 35)		Patients with leg (without midline) lymphoedema (n = 74)		P-value
	n	Mean (±SD)/ median (Q1;Q3)/ number (%)	n	Mean (±SD)/ median (Q1;Q3)/ number (%)	n	Mean (±SD)/ median (Q1;Q3)/ number (%)	
*Patient-related*:							
Age (in years) ^a1^	109	68.0 (±7.3)	34	66.1 (±7.4)	72	68.9 (±7.1)	0.061
BMI (in kg/m^2^) ^a1^	79	27.6 (±3.9)	31	26.6 (±3.2)	48	28.2 (±4.3)	0.071
Smoking status	102		31		71		0.146
Yes		13 (12.7%)		6 (19.4%)		7 (9.9%)	
No		89 (87.3%)		25 (80.6%)		64 (90.1%)	
Missing		7		4		3	
Working status	105		34		71		0.702
Working		30 (28.6%)		8 (23.5%)		22 (31.0%)	
Not working		11 (10.5%)		5 (14.7%)		6 (8.5%)	
Retired		64 (61.0%)		21 (61.8%)		43 (60.6%)	
Missing		4		1		3	
Sport status	106		35		71		0.473
Yes		62 (58.5%)		20 (57.1%)		42 (59.2%)	
No		44 (41.5%)		15 (42.9%)		29 (40.8%)	
Missing		3		0		3	
Medical history:							
Kidney disease	107		34		73		0.726
Yes		10 (9.3%)		4 (11.8%)		6 (8.2%)	
No		97 (90.7%)		30 (88.2%)		67 (91.8%)	
Missing		2		1		1	
Thyroid disease	107		34		73		0.824
Yes		4 (3.7%)		1 (2.9%)		3 (4.1%)	
No		103 (96.3%)		33 (97.1%)		70 (95.9%)	
Missing		2		1		1	
Diabetes	104		32		72		0.316
Yes		10 (9.6%)		4 (12.5%)		6 (8.3%)	
No		94 (9.4%)		28 (87.5%)		66 (91.7%)	
Missing		5		3		2	
Cardiac disease	108		34		74		0.225
Yes		18 (16.6%)		4 (11.8%)		14 (18.9%)	
No		90 (83.3%)		30 (88.2%)		60 (81.0%)	
Missing		1		1		0	
Deep venous thrombosis	108		34		74		0.292
Yes		13 (12.0%)		5 (14.7%)		8 (10.8%)	
No		95 (88.0%)		29 (85.3%)		66 (89.2%)	
Missing		1		1		0	
Chronic venous insufficiency	109		35		74		0.790
Yes		33 (30.3%)		10 (28.6%)		23 (31.1%)	
No		76 (69.7%)		25 (71.4%)		51 (69.7%)	
Missing		0		0		0	
Trauma	103		33		70		0.223
Yes		6 (5.8%)		0 (0.0%)		6 (8.6%)	
No		97 (94.2%)		33 (100%)		64 (91.4%)	
Missing		6		2		4	
Other type of surgery in/nearby affected area which has an influence on the lymphatic transport of the leg and/or midline	104		34		70		0.710
Yes		25 (24.0%)		7 (20.6%)		18 (25.7%)	
No		79 (76.0%)		27 (79.4%)		52 (74.3%)	
Missing		5		1		4	
*Cancer-related*:							
Oncological treatment: Lymph node dissection ^a2^	105		33		72		0.445
Yes		98 (93.3%)		32 (97.0%)		66 (91.7%)	
No		7 (6.7%)		1 (3.0%)		6 (8.3%)	
Missing		4		2		2	
Hormone therapy	103		33		70		0.378
Yes		34 (33.0%)		14 (42.4%)		20 (28.6%)	
No		69 (67.0%)		19 (57.6%)		50 (71.4%)	
Missing		6		2		4	
Radiotherapy ^a2^	105		34		71		0.560
Yes		48 (45.7%)		18 (52.9%)		30 (42.3%)	
No		57 (54.3%)		16 (47.1%)		41 (57.7%)	
Missing		4		1		3	
*Lymphoedema-related*:							
Onset lymphoedema (months) after surgery or LND	85	3.1 (3.1;10.4)	29	3.0 (0.1;8.1)	56	3.9 (0.0;10.9)	0.896
Duration lymphoedema (months) at first consultation	106	26.8 (9.2;54.7)	35	22.2 (8.4;42.4)	68	29.1 (10.9;62.3)	0.182
Locations of lymphoedema:	109		35		74		0.518
Foot							
Yes		64 (58.7%)		19 (54.3%)		45 (60.8%)	
No		45 (41.3%)		16 (45.7%)		29 (39.2%)	
Missing		0		0		0	
Lower leg ^a1^							0.007*
Yes		97 (89.0%)		27 (77.1%)		70 (94.6%)	
No		12 (11.0%)		8 (22.9%)		4 (5.4%)	
Missing		0		0		0	
Upper leg ^a1^							0.026*
Yes		82 (75.2%)		31 (88.6%)		51 (68.9%)	
No		27 (24.8%)		4 (11.4%)		23 (31.1%)	
Missing		0		0		0	
Whole leg with foot							0.144
Yes		108 (99.1%)		34 (97.1%)		74 (100.0%)	
No		1 (0.9%)		1 (2.9%)		0 (0.0%)	
Missing		0		0		0	
Pitting oedema	108		35		73		0.721
Pitting		87 (80.6%)		29 (82.9%)		58 (79.5%)	
Non-pitting		21 (19.4%)		6 (17.1%)		15 (20.5%)	
Missing		1		0		1	
Fibrosis ^a1^	109		35		74		0.034*
Yes		24 (22.0%)		12 (34.3%)		12 (16.2%)	
No		85 (78.0%)		23 (65.7%)		62 (83.8%)	
Missing		0		0		0	
Wounds ^a1^	107		33		74		0.029*
Yes		6 (5.6%)		0 (0.0%)		6 (8.1%)	
NoMissing		101 (94.3%)2		33 (100.0%)2		68 (91.9%)0	
Pain	63		23		40		0.324
Yes		15 (23.8%)		7 (30.4%)		8 (20.0%)	
No		48 (76.2%)		16 (69.6%)		32 (80.0%)	
Missing		46		12		34	
Burden (NRS with 0 no pain – 10 worst pain)	21	4.81 (±3.2)	9	5.9 (±2.8)	12	4.0 (±3.3)	0.188
History of erysipelas	102		35		67		0.169
Yes		14 (13.7%)		5 (14.3%)		9 (13.4%)	
No		88 (86.3%)		30 (85.7%)		58 (78.4%)	
Missing		7		0		7	
Lymphoedema stage	104		34		70		0.382
Stage 0		0 (0.0%)		0 (0.0%)		0 (0.0%)	
Stage 1		29 (27.9%)		11 (32.4%)		18 (25.7%)	
Stage 2 early		60 (57.7%)		16 (47.1%)		44 (62.9%)	
Stage 2 late		15 (14.4%)		7 (20.6%)		8 (11.4%)	
Stage 3		0 (0.0%)		0 (0.0%)		0 (0.0%)	
Missing		5		1		4	
*Lymphoedema treatment-related*:							
Lymphatic surgery ^a1^	106		35		71		0.020*
Yes		3 (2.8%)		3 (8.6%)		0 (0.0%)	
No		103 (97.2%)		32 (91.4%)		71 (100.0%)	
Missing		3		0		3	
Self-management:Bandaging ^a1^	105		33		72		0.082
Yes		22 (21.0%)		11 (33.3%)		11 (15.2%)	
No		83 (79.0%)		22 (66.7%)		61 (84.7%)	
Missing		4		2		2	
Compression garments	108		34		74		0.342
Yes		85 (78.7%)		27 (79.4%)		58 (78.4%)	
No		23 (21.3%)		7 (20.6%)		16 (21.6%)	
Missing		1		1		0	
Skin care	106		33		73		0.309
Yes		58 (54.7%)		20 (60.6%)		38 (52.1%)	
No		48 (45.3%)		13 (39.4%)		35 (47.9%)	
Missing		3		2		1	
Manual lymph drainage ^a1^	105		33		72		0.045*
Yes		9 (8.6%)		6 (18.2%)		3 (4.2%)	
No		96 (91.4%)		27 (81.8%)		69 (95.8%)	
Missing		4		2		2	
Exercises	107		34		73		0.258
Yes		45 (42.1%)		18 (52.9%)		27 (37.0%)	
No		62 (57.9%)		16 (47.1%)		46 (63.0%)	
Missing		2		1		1	
Number of sessions the past 6 months	39	27.8 (±20.9)	17	26.2 (±20.2)	22	29.0 (±21.8)	0.681
Treatment by home physical therapist:							
Bandaging	105		33		72		0.276
Yes		5 (4.8%)		3 (9.1%)		2 (2.8%)	
No		100 (95.2%)		30 (90.9%)		70 (97.2%)	
Missing		4		2		2	
Skin care	68		23		45		0.112
Yes		2 (2.9%)		2 (8.7%)		0 (0.0%)	
No		66 (97.1%)		21 (91.3%)		45 (100.0%)	
Missing		41		12		29	
Manual lymph drainage	107		34		73		0.384
Yes		44 (41.1%)		17 (50.0%)		27 (37.0%)	
No		63 (58.9%)		17 (50.0%)		46 (63.0%)	
Missing		2		1		1	
Exercises	104		33		71		0.869
Yes		8 (7.7%)		3 (9.1%)		5 (7.0%)	
No		96 (92.3%)		30 (90.9%)		66 (93.0%)	
Missing		5		2		3	
Intermittent pneumatic compression	103		33		70		0.977
Yes		7 (6.8%)		2 (6.1%)		5 (7.1%)	
No		96 (93.2%)		31 (93.9%)		65 (92.9%)	
Missing		6		2		4	
Lymph taping	104		33		71		0.794
Yes		2 (1.9%)		1 (3.0%)		1 (1.4%)	
No		102 (98.1%)		32 (97.0%)		70 (98.6%)	
Missing		5		2		3	

^a1^ variables included in the multivariable analyses based on univariable analyses (*p* < 0.1)

^a2^ variables included in the multivariable analyses based on literature (i.e. risk factors for development of lymphoedema

* significant values

#### Multivariable analyses

The variables included in the multivariable model are indicated in Table 1: ^a1^ if they are included based on the univariable analyses with *p* < 0.1 and ^a2^ if they are proven risk factors for lymphoedema [5, 13].

Based on the binary logistic model, the variables lower leg lymphoedema (*p* = 0.020; OR = 0.19 (0.05–0.77)), skin fibrosis (*p* = 0.017; OR = 3.50 (1.25–9.79)), performing self-bandaging (*p* = 0.027; OR = 3.29 (1.14–9.48)) and self-manual lymphatic drainage (p = 0.041; OR = 5.21 (1.07–25.33)) appear to be predictors of having midline lymphoedema from these multivariable analysis. Results of the model based on the 20% data show similar results for lower leg lymphoedema (*p* = 0.24; OR = 0.23 (0.05–1.01)), skin fibrosis (*p* = 0.076; OR = 3.04 (0.92–10.03)) and performing self-bandaging (*p* = 0.007; OR = 4.70 (1.45–15.26)). A different result was obtained for self-manual lymphatic drainage (*p* = 0.284; OR = 2.61 (0.45–15.13).

### C-statistic

The mean area under the curve (AUC) for the proposed prediction of midline lymphoedema was 0.748 (95% CI [0.645–0.851]) with a standard error of 0.053 (see Additional file 2 and 3). This indicates that the characteristics in the model (combination of not having lower leg lymphoedema, having skin fibrosis, self-bandaging and self-manual lymphatic drainage) are good predictors for having midline lymphoedema. Although, based on the 80%-20% cross-validation, 14 out of 15 patients without midline lymphoedema were correctly predicted whereas 1 was not. Of the 7 patients with midline lymphoedema 3 were correctly associated whereas 4 were not. The AUC for the proposed midline lymphoedema was 0.740 (95%CI [0.624–0.856]) with a standard error of 0.059 (see Additional file 4 and 5). Obviously, the performance of the original model is overrated because the model was used for both development and testing. After cross-validation the overfitting was confirmed by the variable self-manual lymphatic drainage which drops out after cross-validation, underlining the importance of the cross-validation.

## Discussion

This is the first study comparing the characteristics of patients with leg and midline lymphoedema versus patients with only leg lymphoedema after treatment for PCa.

Thirty-five patients (32%) had leg and midline lymphoedema, whereas 74 patients (68%) only had leg lymphoedema. So in the present study, a high proportion of midline lymphoedema among prostate cancer patients with lymphoedema was found. Another study to compare this result with is missing. However, the systematic review of Clinckaert et al found a prevalence rate of midline lymphoedema among all prostate cancer patients of 0–1%. They attributed the low prevalence rate by the use of subjective measurement methods and the lack of standardised assessment tools which in turn contributes to the underreporting of the condition. At the Center for Lymphoedema the healthcare providers might have been more aware of this problem, which might lead to more investigation and in turn the higher prevalence rate. A better training and higher awareness of midline lymphedema among oncologists and other care providers referring patients to lymphedema clinics is also necessary. This will shorten the duration between the onset of the lymphoedema and the date of the consultation in the lymphedema clinic. In the present study the median duration between the onset and the date of the consultation was 2.3 years. In a study about vulvar lymphangioma by Simon et al [14], the median time until consultation for genital lymphoedema is 1.4 years. This long duration in both studies underlines again the embarrassment with the midline lymphoedema patients might have. The characteristics of the subjects in the present study where comparable with these in the study of Neuberger et al. [13] which also investigated lower limb lymphoedema after PCa: 93% and 96% of the subjects received lymph node dissection, BMI was 28 and 27 and age was 68y and 65y, respectively. However they did not make a distinction between leg and midline lymphoedema so a comparison about the proportion of patient’s with leg and midline lymphoedema could not be made.

Not having lower leg lymphoedema, having skin fibrosis, self-bandaging and self-manual lymphatic drainage are more frequently seen in patients with leg and midline lymphoedema compared to patients with only leg lymphoedema. Unfortunately, to our knowledge no other studies are currently available to compare the results of this study with. In the following text, we will discuss the most notable variables.

A BMI of 28 (±3.9) is seen in this sample. Although a relation between a high BMI and the development of lymphoedema of the arm and leg has been described elsewhere [15–17], BMI was not significantly different between the groups with and without midline lymphoedema, nor was it a predictor in the multivariable model. However, data about BMI is missing for 30 patients (28%), with almost all belonging to the leg lymphoedema only group. This may have influenced the mean BMI in the leg lymphoedema group.

Significantly more fibrosis is seen in patients with leg and midline lymphoedema (34%) compared to the group with only leg lymphoedema (16%). As stated by The International Society of Lymphology (ISL) consensus document of 2020 [7], fibrosis develops in more advanced stages of lymphoedema, indicating again a more severe disturbance of the lymphatic system in subjects with midline lymphoedema. In this study, wounds are only seen in patients without midline lymphoedema (8%). This result cannot be explained.

Patients with leg and midline lymphoedema more frequently performed self-manual lymphatic drainage compared to the group with leg lymphoedema (18% vs. 4%). This may indicate that the combination of leg and midline lymphoedema had more impact on the patient’s life, resulting in better self-management. Also, patients who perform self-manual lymphatic drainage might be more afraid of the worsening of their lymphoedema and therefore perform this more frequently. Another explanation is that the patient’s physical therapist advised the patient more often to perform self-manual lymph drainage of the midline region, especially because it is difficult to find adequate compression material for this region. However, whether it results in decrease in volume is not clearly investigated [18]. Self-bandaging refers to the application of bandages to the leg by the patients themselves. One possible explanation for this variable could be that it results in increased congestion of lymphatic fluid to the midline region, potentially exacerbating midline lymphoedema. As stated by Vignes, on the other hand, the few midline compression tools which exist might in turn lead to congestion to the leg. In line with the previous variable, also lymphatic reconstructive surgery was more often performed in patients with leg and midline lymphoedema (9%) than in patients with only leg lymphoedema (0%).

All these treatments could imply that patients with midline lymphoedema experience a greater impact from the condition and seek more treatment compared to those with only lower leg lymphoedema.

### Limitations and strengths

This study has some limitations. The main limitation is the retrospective design with inherent biases [19] such as selection bias. It is possible that only patients with more pronounced midline lymphoedema were evaluated (since not all patients have the courage to talk about this problem with their healthcare provider but also because of the healthcare provider, who may refrain from addressing the issue and often does not inquire about swelling in this sensitive area). Which means that those with mild complaints or subclinical cases may have been underrepresented or entirely missed. This might in turn lead to an underestimation of the severity and prevalence of midline lymphoedema. Also, retrospective studies depend on previously recorded data, which may be incomplete or inaccurate. Lastly, objective assessment of midline lymphoedema was not conducted. The problem is that currently there is a paucity of literature on objective measurement methods for midline lymphoedema, leading to the prevalent reliance on subjective inspection in clinical practice. The use of subjective methods to assess midline lymphoedema introduces variability in measurements. Additionally, the interpretation of subjective measurements can be influenced by the observer’s expectations or prior knowledge.

These limitations suggest that the findings of the study may predominantly reflect the characteristics of patients with more severe midline lymphoedema. Potentially missing those with mild midline lymphoedema. Moreover, as indicated by Vignes and Noble-Jones et al. [20], midline lymphoedema is often an embarrassing disorder of which healthcare providers have limited knowledge. Therefore this study in which variables who are associated with presence of midline lymphoedema are shown, is a good start to the investigation of midline lymphoedema.

Nevertheless, the standardised source documents with which all data is collected, and which is the same form that is completed during different consultations is a strength of this study. Another strength is that our study sample is representative for all PCa patients with lymphoedema.

### Clinical implications and future research

This study presents potential characteristics that could serve as predictors of midline lymphoedema. For clinical practice, we recommend to inspect the midline region for presence of lymphoedema when the patient presents without swelling of the lower leg and has skin fibrosis, performs self-manual lymphatic drainage or self-bandaging.

Furthermore, we suggest conducting circumference measurements of the penis and the pitting test and skinfold test at the groin and suprapubic region. For the scrotum a ventral-dorsal circumference measurement can be performed. However, standardized measurement of the scrotum faces difficulties such as the cremaster reflex, thermoregulation and hairiness. We recommend to measure the patient in a supine position which offers more comfort and space for an accurate measurement of the scrotum. We are currently assessing the reliability and validity of these objective measurements in a prospective study.

Additionally, further prospective research is needed to identify predictive variables for the presence of midline lymphedema. Also, studies are needed to explore more suitable compression methods, for the suprapubic region amongst others, and to evaluate the added value of MLD on midline lymphoedema. Additionally, we encourage the investigation of longitudinal outcomes to understand the progression of midline lymphoedema over time and its long-term impact on quality of life.

## Conclusion

This study is the first study comparing the characteristics of patients with leg and midline lymphoedema to patients with only leg lymphoedema. Patients with leg and midline lymphoedema tend to have less frequently lower leg lymphoedema, have more frequently skin fibrosis and perform more frequently self-manual lymphatic drainage and self-bandaging compared to patients with only leg lymphoedema. This indicates that when one of these variables are seen in men with lymphoedema, developed after treatment for PCa, midline lymphoedema is possibly present and should be examined.

Funding and/or Competing interests.

Additional files:

Additional file 1.pdf: Overview of all variables and the method to determine the variable out of the raw data.

Additional file 2.pdf: Area under the curve for the proposed prediction.

Additional file 3.pdf: Performance of the multivariable model to predict midline lymphoedema.

- Support statement: ADG and TDV are a post-doctoral research fellow of the FWO-Flanders.

## Electronic supplementary material

Below is the link to the electronic supplementary material.

Supplementary Material 1

Supplementary Material 2

Supplementary Material 3

Supplementary Material 4

Supplementary Material 5

## Data Availability

The datasets used and/or analysed during the current study are available from the corresponding author on reasonable request.
