# Supplementary material for: Characteristics of lymphoedema, in particular midline lymphoedema, after treatment for prostate cancer: a retrospective study
Source: BMC Urol. 2024 Sep 4;24:192. doi: 10.1186/s12894-024-01533-5 (PMC11373232; doi:10.1186/s12894-024-01533-5)
Supplement: Supplementary file 1 — Supplementary Material 1 [file 12894_2024_1533_MOESM1_ESM.pdf]

Additional file 1

Characteristics of lymphoedema, in particular midline lymphoedema, after treatment for prostate cancer: a retrospective study

Charlotte Van Calster[1], Wouter Everaerts[2,3], Inge Geraerts[1,4], An De Groef[1,5], An-Kathleen Heroes[1], Tessa De Vrieze[1,5], Nele Devoogdt[1,6]

[1]KU Leuven - University of Leuven, Department of Rehabilitation Sciences, Leuven, Belgium

[2]University Hospitals Leuven, Department of Urology, Leuven, Belgium

[3]KU Leuven - University of Leuven, Department of Cellular and Molecular Medicine, Leuven, Belgium

[4]University Hospitals Leuven, Department of Physical Medicine and Rehabilitation, Leuven, Belgium

[5]University of Antwerp, Department of Rehabilitation Sciences and Physiotherapy, MOVANT, Wilrijk, Belgium

[6]University Hospitals Leuven, Center for Lymphedema, Leuven, Belgium

Corresponding author:
Nele Devoogdt
e-mail address: nele.devoogdt@kuleuven.be

Additional file 1: Overview of the different variables and method to determine the variable out of raw data.

**Variable**	**Raw data field(s)**	**Method of determining variable**
Patient-related:		
Age (in years)	Age	NA
BMI (in kg/m²)	BMI	NA
Smoking status (yes/no)	Smoking (yes/no)	NA
Professional status (working/ not working/retired)	Professional status (working/ not working/retired)	NA
Sport status (yes/no)	Sports (yes/no)	NA
Medical history:		
Kidney disease (yes/no)	Kidney disease (yes/no)	NA
Thyroid disease (yes/no)	Thyroid disease (yes/no)	NA
Diabetes (yes/no)	Diabetes (yes/no)	NA
Cardiac disease (yes/no)	Cardiac disease (yes/no)	NA
Thrombosis (yes/no)	Thrombosis (yes/no)	NA
Chronic venous insufficiency (yes/no)	Varicose veins (yes/no); insufficiency proven on venous duplex (yes/no)	If varicose veins = yes OR insufficiency proven on venous duplex = yes
Trauma (yes/no)	Trauma (yes/no)	NA
Surgery beyond surgery for prostate cancer which has an influence on the lymphatic transport of the leg and/or midline (yes/no)	Surgery (yes/no); surgery location (open field); surgery reason (open field)	If surgery, beyond surgery for treating the cancer, at level of leg or pelvic/abdominal region = yes
Cancer-related:		
Oncological lymph node dissection (yes/no)	Oncological lymph node dissection (yes/no)	Oncological lymph node dissection (yes/no)

**Variable**	**Raw data field(s)**	**Method of determining variable**

Oncological hormonal therapy (yes/no)	Oncological hormonal therapy (yes/no)	Oncological hormonal therapy (yes/no)
Oncological radiotherapy (yes/no)	Oncological radiotherapy (yes/no)	Oncological radiotherapy (yes/no)
Lymphoedema-related:		
Onset lymphoedema (in months) after surgery for prostate cancer or gland removal	Surgery for prostate cancer (determined by date of surgery or date of lymph node dissection); onset lymphoedema (date)	Number of days between onset of lymphoedema and surgery, divided by 30
Duration of lymphoedema (in months) at first consultation	Onset lymphoedema (date); consultation date (date)	Number of days between consultation and onset of lymphoedema, divided by 30
Region of leg lymphoedema (unilateral/bilateral)	Based on the different locations (foot, lower leg, upper leg, suprapubic, scrotum) of lymphoedema (yes/no)	If lymphoedema left and right = bilateral OR if lymphoedema only left or right = unilateral
Midline lymphoedema (yes/no)	Penis (yes/no); Suprapubic (yes/no); Scrotum (yes/no)	IF Penis = yes OR Suprapubic = yes OR Scrotum = yes
Location of leg lymphoedema:		
Foot (yes/no)	Left and/or right toes (yes/no); Left and/or right feet (yes/no)	Left and/or right toes = yes OR left and/or right feet = yes
Lower leg (yes/no)	Left and/or right ankle (yes/no); Left and/or right lower leg (yes/no)	Left and/or right ankle = yes OR left and/or right lower leg = yes
**Variable**	**Raw data field(s)**	**Method of determining variable**

Upper leg (yes/no)	Left and/or right upper leg (yes/no); Left and/or right groin (yes/no)	Left and/or right upper leg = yes OR left and/or right groin = yes
Whole leg without foot (yes/no)	Upper leg (yes/no); Lower leg (yes/no)	If upper leg = yes AND lower leg = yes
Whole leg with foot (yes/no)	Foot (yes/no); lower leg (yes/no); upper leg (yes/no)	If Foot = yes AND lower leg = yes AND upper leg = yes
Location of midline lymphoedema:		
Penis (yes/no)	Penis (yes/no)	NA
Suprapubic (yes/no)	Left and/or right abdomen (yes/no); Left and/or right suprapubic (yes/no)	Left and/or right abdomen = yes OR left and/or right suprapubic = yes
Scrotum (yes/no)	Left and/or right scrotum (yes/no)	Left and/or right scrotum = yes
Pitting edema (pitting/non-pitting)	Palpation pitting edema (pitting/non-pitting)	NA
Fibrosis (yes/no)	Palpation fibrosis (yes/no)	NA
Wounds (yes/no)	Skin erosion (yes/no); skin ulceration (yes/no)	If skin erosion = yes OR skin ulceration = yes
Pain (yes/no)	Pain (yes/no)	NA
Burden (scale 0-10)	Scale from 0 to 10	NA
History of erysipelas (yes/no)	History of erysipelas (yes/no)	NA
Lymphoedema stage (stage 0/stage 1/stage 2a/stage 2b/stage 3)	Lymphoedema stage (stage 0/stage 1/stage 2a/stage 2b/stage 3)	NA

Variable	Raw data field(s)	Method of determining variable
Lymphoedema treatment-related		
Lymphatic surgery (yes/no)	Surgical liposuction (yes/no); lymph transplantation (yes/no); lymph-venous anastomoses (yes/no)	If surgical liposuction = yes OR lymph transplantation = yes OR lymph-venous anastomoses = yes
Self management:		
Skin care(days a month)	Skin care(days a month)	NA
Exercises (days a month)	Exercises (days a month)	NA
Bandaging(days a month)	Bandaging(days a month)	NA
Compression garments (days a month)	Compression garments (days a month)	NA
Total number of sessions the past 6 months (days)	Total number of sessions the past 6 months (days)	NA
Treatment by home physical therapist:		
Exercise therapy (yes/no)	Exercise therapy (yes/no)	NA
Manual Lymph Drainage (yes/no)	Manual Lymph Drainage (yes/no)	NA
Bandaging (yes/no)	Bandaging (yes/no)	NA
Intermittent pneumatic compression (yes/no)	Intermittent pneumatic compression (yes/no)	NA
Skin care (yes/no)	Skin care (yes/no)	NA
Physiotherapeutic treatment (last 6 months) (yes/no)	Physiotherapeutic treatment (last 6 months) (yes/no)	If treatment at home = yes

Summary of the variables. NA = not applicable, Raw data field(s) = variable, BMI = Body Mass Index.
