# Supplementary material for: Characteristics of lymphoedema, in particular midline lymphoedema, after treatment for prostate cancer: a retrospective study
Source: BMC Urol. 2024 Sep 4;24:192. doi: 10.1186/s12894-024-01533-5 (PMC11373232; doi:10.1186/s12894-024-01533-5)

Additional file 2

Characteristics of lymphoedema, in particular midline lymphoedema, after treatment for prostate cancer: a retrospective study

Charlotte Van Calster[1], Wouter Everaerts[2,3], Inge Geraerts[1,4], An De Groef[1,5], An-Kathleen Heroes[1], Tessa De Vrieze[1,5], Nele Devoogdt[1,6]

[1]KU Leuven - University of Leuven, Department of Rehabilitation Sciences, Leuven, Belgium

[2]University Hospitals Leuven, Department of Urology, Leuven, Belgium

[3]KU Leuven - University of Leuven, Department of Cellular and Molecular Medicine, Leuven, Belgium

[4]University Hospitals Leuven, Department of Physical Medicine and Rehabilitation, Leuven, Belgium

[5]University of Antwerp, Department of Rehabilitation Sciences and Physiotherapy, MOVANT, Wilrijk, Belgium

[6]University Hospitals Leuven, Center for Lymphedema, Leuven, Belgium

Corresponding author:

Nele Devoogdt

e-mail address: nele.devoogdt@kuleuven.be

Additional file 2: Area under the curve for the proposed prediction

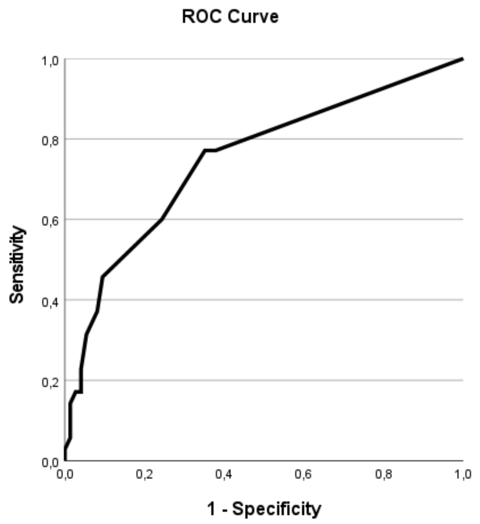

Supplement: Supplementary file 2 — Supplementary Material 2 [file 12894_2024_1533_MOESM2_ESM.pdf]
